# Hand and ultrasonic instrumentation for orthograde root canal treatment of permanent teeth

**DOI:** 10.1590/S1678-77572010000300013

**Published:** 2010

**Authors:** Vinícius PEDRAZZI, Jeronimo Manço de OLIVEIRA-NETO, Patrick SEQUEIRA, Zbys FEDOROWICZ, Mona NASSER

**Affiliations:** 1 DDS, MSc, PhD Associate Professor, Department of Dental Materials and Prosthodontics, Ribeirão Preto Dental School, University of São Paulo, Ribeirão Preto, SP, Brazil.; 2 DDS MSc student, Department of Dental Materials and Prosthodontics, Ribeirão Preto Dental School, University of São Paulo, Ribeirão Preto, SP, Brazil.; 3 BDS, DMD, MSc Specialist, Department of Preventive, Restorative and Pediatric Dentistry, University of Bern, Bern, Switzerland.; 4 BDS, MSc, DPH Director, Bahrain Branch of the UK Cochrane Center, Awali, Bahrain.; 5 DDS, MSc, Researcher, Department of Health Information, Institute for Quality and Efficiency in Healthcare (IQWIG), Cologne, Germany.

**Keywords:** Permanent dentition, Ultrasonic therapy, Root canal therapy

## Abstract

Root canal treatment is a frequently performed dental procedure and is carried out on teeth in which irreversible pulpitis has led to necrosis of the dental pulp. Removal of the necrotic tissue remnants and cleaning and shaping of the root canal are important phases of root canal treatment. Treatment options include the use of hand and rotary instruments and methods using ultrasonic or sonic equipment.

**Objectives:**

The objectives of this systematic review of randomized controlled trials were to determine the relative clinical effectiveness of hand instrumentation versus ultrasonic instrumentation alone or in conjunction with hand instrumentation for orthograde root canal treatment of permanent teeth.

**Material and Methods:**

The search strategy retrieved 226 references from the Cochrane Oral Health Group Trials Register (7), the Cochrane Central Register of Controlled Trials (CENTRAL) (12), MEDLINE (192), EMBASE (8) and LILACS (7). No language restriction was applied. The last electronic search was conducted on December 13th, 2007. Screening of eligible studies was conducted in duplicate and independently.

**Results:**

Results were to be expressed as fixed-effect or random-effects models using mean differences for continuous outcomes and risk ratios for dichotomous outcomes with 95% confidence intervals. Heterogeneity was to be investigated including both clinical and methodological factors. No eligible randomized controlled trials were identified.

**Conclusions:**

This review illustrates the current lack of published or ongoing randomized controlled trials and the unavailability of high-level evidence based on clinically relevant outcomes referring to the effectiveness of ultrasonic instrumentation used alone or as an adjunct to hand instrumentation for orthograde root canal treatment. In the absence of reliable research-based evidence, clinicians should base their decisions on clinical experience, individual circumstances and in conjunction with patients’ preferences where appropriate. Future randomized controlled trials might focus more closely on evaluating the effectiveness of combinations of these interventions with an emphasis on not only clinically relevant, but also patient-centered outcomes.

## INTRODUCTION

Root canal treatment is a procedure that is very frequently performed in dentistry with the aim of retaining teeth. As a treatment option, it offers an alternative to tooth extraction and is carried out on teeth in which irreversible pulpitis has led to necrosis of the dental pulp^11^.

Orthograde root canal treatment entails drilling into the pulp chamber of the tooth which contains the dental pulp. The pulp, which may be inflamed or necrotic, is removed and the root canal is then cleaned and prepared. The objectives of root canal treatment are the elimination of infection from the root canal and the prevention of its reinfection by the filling and sealing of the root canal space^5,16^.

It is generally recognized that a cleaner root canal system should lead to improved outcomes^7^ and that successful root canal treatment may prolong the retention of the tooth as a functional unit in the mouth.

Some of the problems encountered in the cleaning and shaping of root canals have led to a wide search for innovative materials, instruments and techniques which might permit a faster and more effective way of achieving a disinfected and debris-free canal that is ready for obturation. Apart from the traditional methods of using hand and rotary instruments, more recent techniques have employed lasers, non-instrumentation techniques (NIT), and methods using ultrasonic or sonic equipment. A number of studies have shown that endodontic files which have been activated by ultrasonic energy may be effective in both the cleaning and shaping of root canal systems^18^.

Hand instrumentation is the traditional method of preparation of root canals and involves the use of files to clean and shape the root canals with the aim of removing pulpal tissue, infected debris and some of the inner, infected pulpal dentine. Copious irrigation to flush the canal accompanies this instrumentation. The second aim is to shape the canal in such a way that it can be filled completely to prevent the canal becoming further infected by microorganisms.

Using ultrasonic devices in addition to hand instrumentation presupposes that benefits may accrue and outcomes may be improved when compared to hand instruments alone. Ultrasound is sound energy with a frequency over 20,000 oscillations *per* second. The first commercial machine designed for cleaning and disinfecting the root canal was introduced 30 years ago^14^. This process involves the activation of a file with ultrasound which can then be used to both clean and shape the dentine of the root canal. Ultrasound instrumentation can be used either as a primary cleaning and shaping technique or after hand instrumentation. These two techniques require the active movement by the operator of the ultrasonic instrument against the canal walls. Alternatively, ultrasonic energy can be applied passively, without any contact with the canal walls and without any movement of the instrument after activation is started^10^. It was previously believed that it was necessary to move the file in the canal. However, more recent microscopic observation suggests that it is only necessary to bring ultrasonic energy into the canal, and that even a straight, blunt passive wire will transmit enough energy to clean the canal further.

A well recognized difficulty that can arise during ultrasonic preparation is the accurate control of the cutting effect of the file^19^. Ultrasonic instrumentation may also result in lengthier treatment time, and the operational and maintenance requirements of the ultrasonic equipment may add substantially to the treatment cost.

Hand instrumentation of root canals requires irrigation to remove the debris produced. This irrigation may be carried out with fine syringes introduced into the canal orifice. Alternatively, ultrasonic irrigation of root canals can be performed with or without simultaneous ultrasonic instrumentation^20^.

Successful root canal treatment is characterized by the absence of symptoms and clinical signs and any radiographic signs of periodontal involvement^6^. The success of orthograde root canal treatment depends on a series of variables some related to the pre-operative conditions of the tooth as well as the endodontic procedures^5^ , with curved canals posing possibly some of the most significant challenges. Whilst it is perceived that the improved cleaning that occurs with ultrasonic instruments may lead to improved outcomes for endodontically treated teeth, complications may arise. Complications can be broadly divided into four categories:

(1) Blockage, ledging, and loss of working length in the canal

(2) Deviations from the normal canal or root anatomy

(3) Excessive or inadequate canal preparation

(4) Breakage of instruments in the canal.

Root canal treatment has a good degree of success (approximately 80%)^12^. However, root canal treatment can fail, usually due to technical reasons. even when technical excellence is attained, failure may still ensue from remaining infection, because of the nature of the root canal and the inability of current methods to completely clean and fill all its niches^15^. If ultrasonic instrumentation is able to more effectively clean the root canal then it may be expected to result in improved treatment outcomes.

Ultrasonic irrigation of the root canals can be performed with or without simultaneous ultrasonic instrumentation. Passive ultrasonic irrigation (PUI) can be an important supplement for cleaning the root canal system and, compared with traditional syringe irrigation, is capable of removing more organic tissue, bacteria and dentin debris from the root canal system. It has been claimed that PUI is more efficient in cleaning canals than ultrasonic irrigation with simultaneous ultrasonic instrumentation^20^.

The objectives of this systematic review of randomized controlled trials were to determine the relative clinical effectiveness of hand instrumentation versus ultrasonic instrumentation alone or in conjunction with hand instrumentation for orthograde root canal treatment of permanent teeth.

## MATERIAL AND METHODS

A systematic review of randomized controlled trials comparing hand instrumentation versus ultrasonic instrumentation alone or as and adjunctive procedure to hand instrumentation for orthograde root canal treatment of permanent teeth was undertaken. Only trials with adult participants (≥18 years old) with single and multiple permanent teeth with completely formed apices, and no evidence of internal resorption requiring root canal treatment were included in the review. Patients undertaking re-treatment of a tooth were excluded. The outcomes included were as follows:

### Primary outcomes

(1) Proportion of teeth retained for at least 12, 24, 36 and 48 months and their periapical status as confirmed by radiograph.

(2) Total time required for preparation technique and number of visits.

(3) Postoperative pain: self assessment of pain measured on a visual analogue scale or similar, use of pain medication and antibiotic medicine (type, dosage and amount).

### Secondary outcomes

(1) Any unscheduled re-visit or emergency visit.

(2) Any quality of life or patient satisfaction outcomes measured on a validated scale.

For the identification of studies included or considered for this review, detailed search strategies were developed for each of the following databases:

The Cochrane Oral Health Group Trials Register (whole database, to December 13^th^, 2007);

The Cochrane Central Register of Controlled Trials (CENTRAL) (The Cochrane Library Issue 4, 2007 – 17 October 2007);

MEDLINE (via OVID) (without filter) (from 1966 to December, 13^th^ 2007);

EMBASE (via OVID) (without filter) (from 1980 to December, 13^th^ 2007);

Latin American and Caribbean Literature on Health Sciences (LILACS) (via BIREME) (without filter and no date limit) (on December, 13th 2007).

The search strategy retrieved 226 (7 Cochrane Oral Health Group Trials Register, 12 CENTRAL, 8 EMBASE, 192 MEDLINE, 7 LILACS) references. All databases were searched up to 13 December 2007. Search strategies were developed for MEDLINE, but were revised appropriately for each database.

The reference lists of the potentially eligible clinical trials and the review authors’ personal databases of trial reports were also searched in an attempt to identify any other relevant studies. There were no language restrictions on included studies and we translated one relevant non-English paper.

The abstracts of studies resulting from the searches were independently assessed by three reviewers (Patrick Sequeira, Zbys Fedorowicz and Jeronimo Manço de Oliveira Neto), and all irrelevant studies were excluded. Full-text reprints of all relevant and potentially relevant studies, that is, those appearing to meet the inclusion criteria, or those that had insufficient information in the title and abstract to make a clear decision, were obtained. The full-text reprints were assessed independently by these three review authors, and any disagreement on the eligibility of included studies was discussed and resolved. Studies not matching the inclusion criteria were excluded from further review, and their details and reasons for their exclusion were recorded.

Although no eligible randomized controlled trials met the inclusion criteria for inclusion in the present investigation, the following methods were to be applied and will be used if further trials are identified for inclusion in any updates of this review.

### Assessment of methodological quality

Grading and assessment of the selected studies was to be done independently by two review authors (Vinícius Pedrazzi and Jeronimo Manço de Oliveira Neto), and according to the criterion grading system described in the *Cochrane Handbook for Systematic Reviews of Interventions* 4.2.6.

### Data collection

Study details and outcomes data were to be collected using a predetermined form designed for this purpose. extracted data were to be entered separately by each of two review authors (Mona Nasser and Patrick Sequeira) into the "Characteristics of included studies" table in RevMan 4.2, and were automatically checked for differences. Data would only be included if there was an independently reached consensus. Zbys Fedorowicz held the master copy of the review.

The following details were to be extracted.

(1) Study methods: method of allocation, masking of participants and outcomes, exclusion of participants after randomization and proportion of follow-up losses;

(2) Participants: country of origin of the study, sample size, age, gender, inclusion and exclusion criteria;

(3) Intervention: duration and length of time in follow-up;

(4) Control: either of the two interventions used as a control;

(5) Outcomes: as described in the section on outcome measures.

This information was to be used to help assessing the heterogeneity and the external validity of the trials.

### Findings

The search strategy retrieved 226 (7 Cochrane Oral Health Group Trials Register, 12 CENTRAL, 8 EMBASE, 192 MEDLINE, 7 LILACS) references to studies, which were independently assessed for relevance by three of the review authors (Patrick Sequeira, Zbys Fedorowicz and Jeronimo Manço de Oliveira Neto). Only 10 references^1,2,3,4,8,9,13,17,21,22^ were considered for further analysis.

Full-text reprints of these 10 remaining studies were obtained. Their reference lists were examined, but they did not provide any additional citations to potentially eligible studies. We arranged to translate the studies that were written in Chinese, Italian, Russian and the Japanese languages. None of the retrieved studies, however, met our inclusion criteria and were excluded from the present review. The reasons for their exclusion were noted. See Figure 1. In summary, no relevant randomized controlled trials were found for this review and therefore no data were available.

**Figure 1:** Characteristics of excluded studies

**Study**	**Reason for exclusion**
Burleson^11^(2007)	The trial did not evaluate any of the primary or secondary outcomes of this review
Carli^2^(1989)	*In vitro* study
Carver^3^(2007)	The trial did not evaluate any of the primary or secondary outcomes of this review
Chan^4^(1990)	*In vitro* study
Hong^8^(1998)	Non-randomized study
Ishikawa^9^(1988)	Non-randomized study
Makeeva^13^(2005)	*In vitro* study
Palazzo^17^(1989)	Review. Non-clinical study
Wu^21^(1993)	Comparison of canal irrigants
Xiong^22^(2001)	Comparison of irrigants

## DISCUSSION

Successful endodontic treatment is largely dependent on the complete removal of all necrotic tissue remnants and on the overall reduction in number of bacterial organisms in the root canal. Careful preparation, shaping and subsequent obturation of the root canal are essential steps in the process.

However, due to the complex nature and irregularity of root canal anatomy, the process of cleaning and shaping can be very time consuming and laborious. Although ultrasonic instrumentation for root canal treatment would appear to offer several advantages over the traditional method of hand instrumentation, the use of ultrasonically driven instruments has not been universally accepted.

The majority of studies that were examined had been conducted on extracted teeth, and the only retrieved clinical trials comparing ultrasonic and hand instrumentation had assessed issues that were not within the scope of this review^1,3^. This noticeable absence of trials highlights the need for investigators in future trials to ensure they identify and report not only clinically relevant outcomes, but also those that are of importance to patients.

Many of the trials that were examined in this review compared the cutting efficiency and other characteristics of hand files with ultrasonically activated files, and the most frequently reported outcomes were expressed as debris indices, sterility and bacterial counts and overall cleanliness of prepared canals. However, the general perception of ultrasonic instrumentation as a major technological advancement in endodontics, and its apparent superiority for primary instrumentation of root canals does not appear to have been confirmed in these trials.

Many of the limitations, as well as some of the possible applications, of ultrasonic instrumentation used alone for root canal treatment are well recognized, but there appears to be a need for further research that focuses on ways in which these applications, in particular improved debridement in less accessible canals, can be used as an adjunct to hand instrumentation.

The results of this systematic review confirm that future research should include more *in vivo* trials with outcomes that are patient-centered as listed in the primary outcomes for this review, and trials that are robust, well designed and reported according to the CONSORT statement (available from http://www.consortstatement.org/).

## CONCLUSIONS

This review illustrates the current lack of published or ongoing randomized controlled trials and the unavailability of high-level evidence based on clinically relevant outcomes referring to the effectiveness of ultrasonic instrumentation used alone or as an adjunct to hand instrumentation for orthograde root canal treatment. In the absence of reliable research-based evidence, clinicians should base their decisions on clinical experience, individual circumstances and in conjunction with patients’ preferences where appropriate. Future randomized controlled trials might focus more closely on evaluating the effectiveness of combinations of these interventions with an emphasis on not only clinically relevant, but also patient-centered outcomes.
